# In Vitro and In Vivo Characterization of Novel Cathelicidin-Based Peptides with Antimicrobial Activity Against *Pseudomonas aeruginosa*

**DOI:** 10.3390/antibiotics14080838

**Published:** 2025-08-19

**Authors:** Javier Moreno-Morales, Núria Martín-Vilardell, Salvador Guardiola, Xavier Vila-Farrés, Tania Cebrero, Marko Babić, Clara Ballesté-Delpierre, Daniela Kalafatović, Ernest Giralt, María Eugenia Pachón-Ibañez, Jordi Vila

**Affiliations:** 1 Barcelona Institute for Global Health (ISGlobal), 08036 Barcelona, Spain; 2 Department of Basic Clinical Practice, Faculty of Medicine and Health Sciences, University of Barcelona, 08036 Barcelona, Spain; 3 Institute for Research in Biomedicine (IRB Barcelona), The Barcelona Institute of Science and Technology (BIST), 08028 Barcelona, Spain; 4 Institute of Biomedicine of Seville (IBiS), University Hospital Virgen del Rocío/Spanish National Research Council (CSIC)/University of Seville, 41013 Seville, Spain; 5 University of Rijeka, Faculty of Engineering, 51000 Rijeka, Croatia; marko.babic@uniri.hr (M.B.); daniela.kalafatovic@uniri.hr (D.K.); 6 CIBER in Infectious Diseases (CIBERINFEC), Instituto de Salud Carlos III, 28029 Madrid, Spain; 7 Department of Inorganic and Organic Chemistry, Faculty of Chemistry, University of Barcelona, 08028 Barcelona, Spain; 8 Department of Clinical Microbiology, Hospital Clínic de Barcelona, 08036 Barcelona, Spain

**Keywords:** antimicrobial peptides, AMR, novel antibiotics, bactericidal, CAP-18

## Abstract

**Background/Objectives**: Infections caused by multidrug-resistant (MDR) *Pseudomonas aeruginosa* are steadily increasing, thus the discovery and development of new and effective agents are needed. Antimicrobial peptides (AMPs) are a heterogeneous group of innate defense system peptides with broad antimicrobial activity. In this study, 17 AMPs were tested, identifying CAP-18, a cathelicidin-based compound, as the most active. CAP-18 was optimized by synthesizing structural derivatives, which were selected for further studies based on their activity against a collection of MDR and colistin-resistant *P. aeruginosa* strains. **Methods**: AMPs collection was initially tested against different *P. aeruginosa* strains, identifying CAP-18 as the most active. CAP-18 derivatives were synthetized and assessed by the Minimum Inhibitory Concentration (MIC), time-kill kinetics, cytotoxicity against human cell lines, hemolytic activity, and therapeutic index (IC_50_/MIC_90_). The mechanism of action was assessed by Transmission Electron Microscopy (TEM), and in vivo efficacy was determined through a murine skin infection model. **Results**: CAP-18 and D-CAP-18 had a MIC_90_ of 4 and 2 μg/mL, respectively, whereas CAP-18_31_ and D-CAP-18_31_ presented MIC_90_ values of 16 mg/L. The shorter derivatives of CAP-18 showed a lower activity. Time-kill curves revealed a fast bactericidal effect. These derivatives showed low toxicity against different human cell lines and low hemolysis, resulting in a wide therapeutic index (IC_50_/MIC_90_), with D-CAP-18 having the best therapeutic index (137.4). TEM provided insight into the mechanism of action, revealing bacterial membrane damage. In vivo studies of both CAP-18 and D-CAP-18 showed good activity with a 3 log decrease compared to the infected control group. **Conclusions**: Among the investigated four peptides, D-CAP-18 is the most promising candidate to treat skin infections caused by MDR *P. aeruginosa* since it shows potent activity both in vitro and in vivo, and a high therapeutic index.

## 1. Introduction

The fact that AMR is a current global health threat is proven by recent estimates, which point out that 1.27 million deaths worldwide and 133,000 in the WHO European region were attributable to infections caused by antimicrobial-resistant (AMR) bacteria only in 2019 [1,2]. Despite the implementation of action plans to combat antimicrobial resistance by controlling the emergence and spread of multidrug-resistant bacteria, there remains an urgent need for new antibiotics. However, the larger pharmaceutical companies have left the field because of the increasing cost of drug development and an especially low return on investment in antibiotic discovery and development [3,4]. WHO developed tools like the AWaRe classification, a pathogen priority list, and pipeline reviews, to preserve and guide antibiotic use and development [5,6,7]. *Pseudomonas aeruginosa* is classified into the critical priority category in the WHO’s pathogen priority list. This pathogen is usually responsible for healthcare-associated infections; nosocomial infections by multidrug-resistant (MDR) bacteria turn into longer hospital stays, increased treatment costs, and higher mortality [8]. *P. aeruginosa* is a common agent in healthcare-associated infections, with a prevalence of 7.1 to 7.4% of nosocomial infections, being especially prevalent in intensive care units [8,9,10]. Although the most common site of infection is pneumonia, *P. aeruginosa* also causes surgical site infections, urinary tract infections, and bacteremia. Infections by MDR strains are devastating in pediatric burn intensive care units, being one of the most prevalent etiological agents [11,12].

Several alternative strategies are under investigation for antibacterial discovery, including monoclonal antibodies, phage therapy, antivirulence agents, nanoparticles, antisense oligonucleotides, vaccines, and antimicrobial peptides (AMPs) [13,14,15]. AMPs are innate immune effectors found across multicellular organisms, acting at barrier sites or being rapidly produced in response to infection [16,17]. They exhibit broad-spectrum activity against bacteria, viruses, fungi, and yeast [18,19], with a lower likelihood of inducing resistance due to their rapid and non-specific mechanisms [20,21]. Typically, 10–50 amino acids long, AMPs are rich in hydrophobic, hydrophilic, and cationic residues, which facilitate membrane binding and disruption via amphipathic interactions [21,22]. The main drawbacks for clinical usage of AMPs are their toxicity, stability, and high production costs [21,23].

Cathelicidins are a diverse family of antimicrobial peptides that vary widely in structure, size, and amino acid sequence. They are predominantly stored in the secretory granules of neutrophils, macrophages, and epithelial cells and are released upon leukocyte activation. Members of this family share a conserved N-terminal cathelin domain, which distinguishes them from other immune peptides. In contrast, their C-terminal antimicrobial domain, typically ranging from 12 to 80 amino acids in length, is highly variable both between and within species. This domain often adopts α-helical or β-hairpin structures and can be enriched in proline and arginine residues, contributing to their functional diversity [24,25,26,27]. Cathelicidins are initially synthesized as inactive prepropeptides and are stored in neutrophil granules; they become biologically active following cleavage by elastases that release the mature peptide [28]. The first antimicrobial peptides identified were insect-derived cecropins [29] and amphibian magainins [30]. Mammalian cathelicidins were discovered later, with rabbit CAP-18 being one of the earliest characterized examples [31,32,33], followed by bovine bactenecins Bac5 and Bac7, and porcine cecropin P1 [25,34,35].

CAP-18 (Cathelicidin Antimicrobial Peptide 18) is a cationic antimicrobial peptide originally isolated from rabbit granulocytes. It is synthesized as an 18 kDa precursor protein consisting of 142 amino acids, comprising a conserved cathelin-like domain and a C-terminal antimicrobial domain. Upon proteolytic processing, the mature, active form is released as a 37-residue peptide (GLRKRLRKFRNKIKEKLKKIGQKIQGLLPKLAPRTDY), which exhibits potent antimicrobial activity. This peptide has been shown to bind and neutralize lipopolysaccharide (LPS), modulate inflammatory pathways, and protect mice from LPS-induced shock at high concentrations [33,36,37]. Interestingly, while proteolytic processing is typically required for cathelicidin activation, the full-length (proform) CAP-18 also demonstrates biological activity. It can inhibit LPS activity and act synergistically with other host defense molecules, likely due to its net positive charge (+8) [38]. Structurally, CAP-18 adopts a rigid α-helical conformation with distinct cationic and hydrophobic regions that facilitate interactions with bacterial membranes, contributing to its antimicrobial function [39]. By shortening the length of CAP-18 at both the carboxy- and amino-terminal ends, we aimed to identify the minimal amino acid sequence required to retain antimicrobial activity and to assess the functional importance of the removed residues.

This study aims to analyze and optimize the antimicrobial activity of CAP-18 against a collection of *P. aeruginosa* clinical isolates, through the synthesis of peptide analogs derived from its structure, defining the peptides’ in vitro biological profile against human cell lines and erythrocytes, determining its bactericidal activity, obtaining a first approach on the effect of CAP-18 molecules on the bacterial membrane through Transmission Electron Microscopy (TEM) and finally to carry out an investigation of its in vivo effectiveness in a skin infection mouse model by *P. aeruginosa*.

## 2. Results

### 2.1. Antimicrobial Susceptibility Profile

Seventeen peptides from different sources, including α and β defensins and cathelicidins, were tested against two clinical isolates of *P. aeruginosa*, one colistin-susceptible and one colistin-resistant. All tested peptides exhibited a minimum inhibitory concentration (MIC) greater than 32 mg/L, except for CAP-18, which showed MICs of 8 and 4 mg/L against the susceptible and resistant isolates of *P. aeruginosa*, respectively (Table 1).

Therefore, we focused on CAP-18 and its derivatives, which were designed by shortening the 37-mer peptide to lengths of 31, 23, 21, 19, 18, and 14 amino acids through truncation of both N- and C-termini. Among these, only CAP-18_31_ retained antimicrobial activity. The MICs of CAP-18 and CAP-18_31_ for *P. aeruginosa* strain 1211007 and ATCC 1211101 were 1 mg/L (0.2 μM) and <0.125 mg/L (<0.03 μM), and 8 mg/L (2.1 μM) and 0.5 mg/L (0.1 μM), respectively (Table 2).

Since unmodified linear peptides typically show poor in vivo stability [40], we decided to synthesize the enantiomer and retro-enantiomer of CAP-18 and CAP-18_31_, which are entirely composed of D-amino acids, thus rendering them stable to proteolytic degradation. Specifically, the retro-enantio approach aims to recapitulate the side chain topology of the parental L-form by reversing the sequence and inverting the C_α_ chirality [41,42]. The enantiomers (D-CAP-18 and D-CAP-18_31_) showed identical MICs of 2 and 0.25 mg/L for strains 1211007 and ATCC 121110, respectively, while the retro-enantiomers (R-CAP-18 and R-CAP-18_31_) displayed MICs of 2 and 32 mg/L, and 0.5 and 4 mg/L, respectively, against the two aforementioned strains (Table 2).

Retroenantiomers R-CAP-18 and R-CAP-18_31_ showed MICs only a few folds higher than their respective enantiomers and the ‘original’ peptides. This discouraged further screening of these peptides against an extended collection of *P. aeruginosa*. Thus, CAP-18, CAP-18_31_, D-CAP-18, and D-CAP-18_31_ were selected for further antimicrobial activity characterization. To this end, broth microdilution assays were conducted on these four peptides against a panel of 15 bacterial strains of *P. aeruginosa* to obtain MIC_50_ and MIC_90_ values (Table 3 and Table A1). Colistin was included as a comparator due to its peptide nature and status as a last-resort antibiotic in the clinic. Colistin MIC_90_ was 64 mg/L, given the deliberate selection of the strain collection. All four peptides maintained activity against MDR and colistin-resistant strains of *P. aeruginosa*, with MIC_90_ values of 4 and 2 mg/L for CAP-18 and its enantiomer D-CAP-18, respectively, and 16 mg/L each for CAP-18_31_ and its enantiomer D-CAP-18_31_, although their MIC_50_ values were maintained at 2 and 4 mg/L, respectively (Table 3).

### 2.2. Hemolytic and Cytotoxic Effect in Human Cells

**Figure 1:**
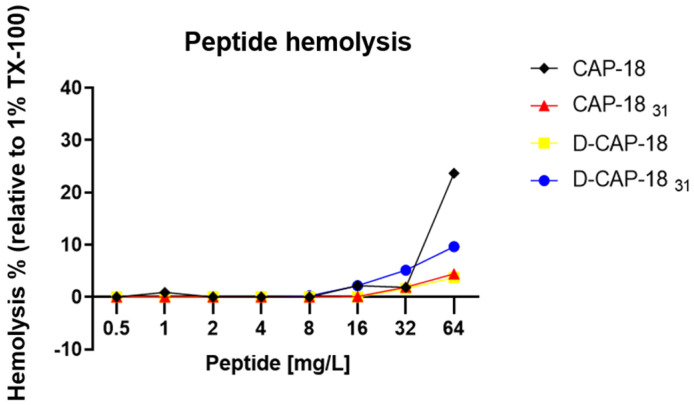
Peptide erythrocyte hemolysis % (relative to 1% TX-100 treatment) versus peptide concentration for CAP-18, CAP-18_31_, D-CAP-18, and D-CAP-18_31_.

One of the main hurdles for the clinical development of AMPs is nonspecific cytotoxicity for human cells. Study of hemolysis and cell viability assays in early preclinical stages allows for the selection of agents with the lowest toxicity from those that presented the most potent antimicrobial activity prior to in vivo assays. Here, the hemolytic effect of CAP-18, D-CAP-18, CAP-18_31_, and D-CAP-18_31_ peptides was measured in vitro against human erythrocytes, using Triton X-100 (TX-100) as a control for total hemolysis (Table 4 and Figure 1). All peptides had hemolysis levels below 10% at concentrations <32 mg/L (around 8 µM), suggesting low toxicity at clinically relevant concentrations (Table 4). The therapeutic index against *P. aeruginosa*, calculated as the ratio of hemolysis IC50 values to MIC_90_ values [43], was highest for D-CAP-18 (137x) and lowest for D-CAP-18_31_ (25x) (Table 4).

**Figure 2:**
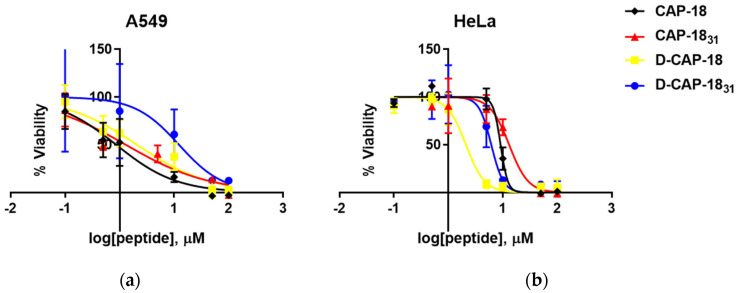
Viability of A549 (**a**) and HeLa human cells (**b**) treated for 24 h with increasing concentrations of CAP-18, CAP-18_31_, D-CAP-18, and D-CAP-18_31_ (for A549) as measured with the XTT viability marker.

On human cell lines, high cytotoxicity was recorded for the peptides against A549 cells (Table 4, Figure 2a). The D-isomers were less toxic than their enantiomers, with IC_50_ values of 2.09 µM for D-CAP-18 and 12.3 µM for D-CAP-18_31_. Toxicity was lower against HeLa cells for all peptides, with CAP-18_31_ and CAP-18 being the least toxic (IC_50_ of 13.1 and 9.12 µM, respectively) (Figure 2b). In contrast, D-CAP-18 was the most toxic peptide against HeLa cells (IC_50_ of 2.11 µM), close to its IC_50_ against A549 cells.

### 2.3. Time-Kill Kinetics Assays

**Figure 3:**
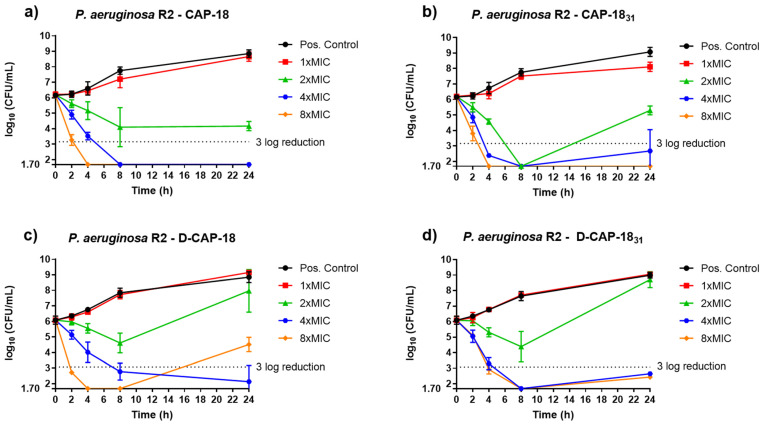
*P. aeruginosa* R2 TKCs against (**a**) CAP-18; (**b**) CAP-18_31_; (**c**) D-CAP-18; and (**d**) D-CAP-18_31_.

**Figure 4:**
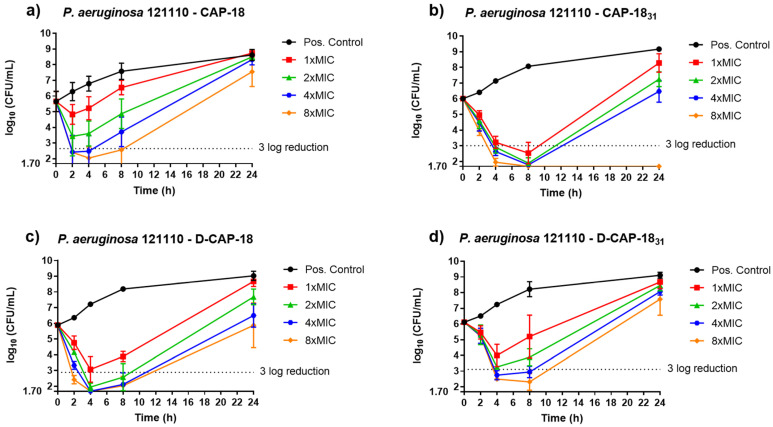
*P. aeruginosa* 121110 TKCs against (**a**) CAP-18; (**b**) CAP-18_31_; (**c**) D-CAP-18; and (**d**) D-CAP-18_31_.

In vitro antibacterial activity of compounds CAP-18, D-CAP-18, CAP-18_31_, and D-CAP-18_31_ against *P. aeruginosa* strains was studied through time-kill curves (TKC) (Figure 3 and Figure 4). CAP-18 reached a bactericidal effect at 4× MIC after 8 h incubation and at 8× MIC at 4 h; both these concentrations were completely bactericidal for strain R2 (colistin-resistant *P. aeruginosa* clinical isolate) as it stayed below the growth detection limit (1.70) after 24 h incubation at 37 °C (Figure 3a). The enantiomer D-CAP-18 also reached a bactericidal effect after 8 h of incubation at 4× MIC, which was maintained after 24 h of incubation. Nonetheless, a 3 log reduction was also observed after 4 h for 8× MIC, but afterwards a regrowth was reported (Figure 3c). The shorter peptide, CAP-18_31_, showed a strong bactericidal effect over time: this peptide was bactericidal at 4 h incubation for both 4× MIC and 8× MIC, and also at 2× MIC at 8 h; however, bacterial regrowth was recorded at all concentrations of the peptide at 24 h incubation except for 8× MIC, which maintained the bactericidal effect after 24 h (Figure 3b). The enantiomer D-CAP-18_31_ showed similar activity for 4× MIC and 8× MIC, reaching cidality after 8 h and 4 h of incubation, respectively, which was maintained after 24 h of incubation (Figure 3d).

For strain *P. aeruginosa* ATCC 121110 (Figure 4), in general, CAP-18, D-CAP-18, CAP-18_31_, and D-CAP-18_31_ showed the same tendency, observing a decrease in the log_10_ CFU/mL after 4 h of incubation at the different established peptide concentrations: MIC, 2× MIC, 4× MIC, and 8× MIC. Regarding CAP-18, 4× MIC and 8× MIC showed a bactericidal effect after 2 h of incubation, maintaining close bacterial concentration until 4 h, which regrew over time (Figure 4a). This fast bactericidal activity was improved when testing CAP-18_31_, which showed a 3 log reduction during the 4 h to 8 h of incubation at all concentrations (MIC, 2×, 4×, and 8×), and a total eradication of the bacterial growth after 24 h of incubation at 8× MIC (Figure 4b). As for D-CAP-18, it acted similarly to its enantiomer, having a reduction in more than 3 log_10_ CFU/mL for 2× and 4× MIC after 4 h of incubation, and after 2 h of incubation for 8× MIC (Figure 4c). Finally, D-CAP-18_31_ decreased bacterial growth up to 3 log after 4 h of incubation at 4× MIC and 8× MIC (Figure 4d). In all cases, bacterial regrowth was observed after 8 h of incubation. For *P. aeruginosa* 121110, all four peptides generally showed a 3 log reduction generally after 4 h to 8 h incubation, but a regrowth after 24 h of incubation. However, CAP-18_31_ was the most active one, having a bactericidal effect after 24 h, having CFU values under the limit of detection.

### 2.4. Bacterial Membrane Damage Through Transmission Electron Microscopy (TEM)

**Figure 5:**
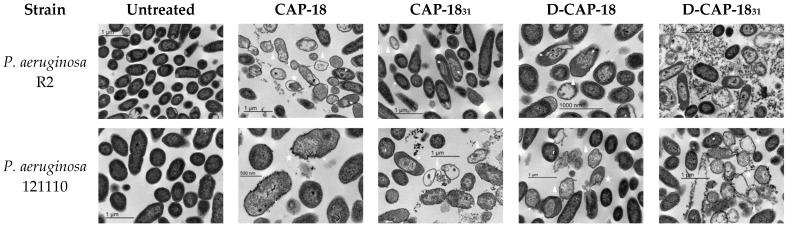
TEM images of *P. aeruginosa* R2 and 121110 after 4 h incubation at 4× MIC. White arrows indicate cytoplasm aggregation; white stars indicate ruptured membranes; white triangles indicate empty membranes.

The effect of CAP-18, CAP-18_31_, D-CAP-18, and D-CAP-18_31_ on cell morphology *P. aeruginosa* R2 and 121110 strains was studied using TEM. *P. aeruginosa* R2 and *P. aeruginosa* 121110 untreated cells showed wrinkly membranes, most probably due to sample chemical fixation and dehydration steps (Figure 5). Fixation artifacts aside, micrographs of peptide-treated bacteria most notably show different stages of cellular damage. Elongated outer membranes with empty inner spaces and inner content aggregation and disintegrating bacterial cells with compromised membranes in different lysis states. In the case of *P. aeruginosa* 121110 in peptide-treated samples, cytoplasm-empty membranes filled with contorted vesicles were seen, different grades of cell lysis were also observed, and bubbles protruding from the cell outer membrane (blebs) were noticed. These blebs are produced by bacteria in stressful conditions, such as antibiotics.

Nonetheless, it has to be highlighted that CAP-18 and derivatives still maintain activity against highly colistin-resistant strains (Appendix A Table A1).

### 2.5. Modeling the Effect of Sequence Truncation on Helical Content

Peptides show a higher conformational flexibility in solution when they are not part of a larger 3D scaffold (the protein). Consequently, it is not straightforward to compare secondary structures of specific regions found in fully folded proteins and those of the de novo synthesized sequences. The mechanism of action of CAP-18 derivatives is based on the assumed helical structure of the peptide [39,44]. Furthermore, the 32–37 amino acid region called the CDR (carboxyl-terminal disordered region) is highly variable and subject to selection mechanisms. Removing different sections of the original sequence might lead to conformational changes and higher flexibility. For this reason, we wanted to estimate the propensity of CAP-18 and its fragments CAP-18_31_, CAP-18_23_, CAP-18_21_, CAP-18_19_, CAP-18_18,_ and CAP-18_14_ to form helical structures using PEP-FOLD4 [45], which allows secondary structure prediction of proteinogenic peptides in physiological solution, and is particularly suited for poly-charged peptides such as CAP-18.

**Figure 6:**
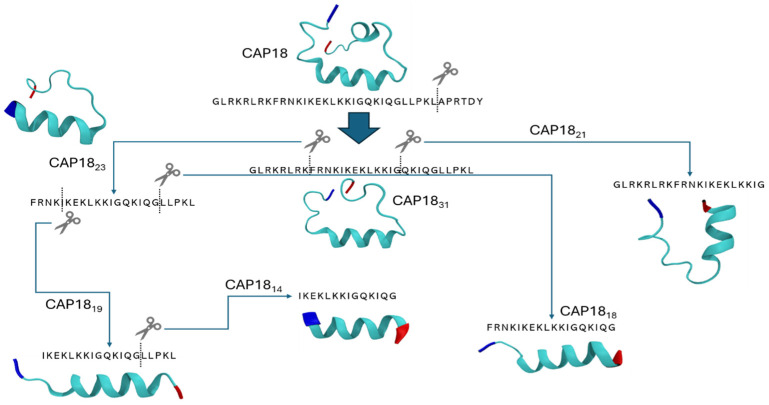
The CAP-18 variants and their conformation based on their best scoring model from PEPFOLD4 for CAP-18, CAP-18_31_, CAP-18_23_, CAP-18_21_, CAP-18_19_, CAP-18_18_, and CAP-18_14_. The N- and C-termini are colored.

**Figure A1:**
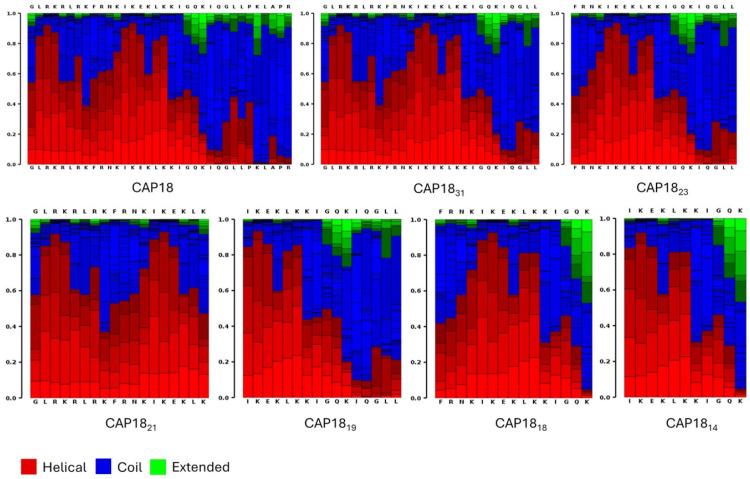
A graphical representation of the probabilities of each structural alphabet letter assuming a certain conformation (vertical axis) at each position of the sequence (horizontal axis), where structural alphabet letters correspond to fragments of 4 residue length. Because of this, the last few letters of each sequence are unavailable.

All the examined derivatives showed helical conformations that are consistently observed in the KLKKI region (Figure 6). The LPKL region either forms helical structures in CAP-18 or coils in its shorter versions. More in detail, the helical regions of CAP-18 are RKFRNKIKEKLKKI and LPKL, similarly to CAP-18_31_ and CAP-18_23_ that had a slightly shorter helical region (FRNKIKEKLKKI), showing significant overlap with CAP-18 (Figure 6). CAP-18_21_ formed a helix in its KIKEKLKKIG region, losing the FRN beginning compared to its longer variants, CAP-18_31_ and CAP-18_23_. CAP-18_19_ presented its helix in the KIKEKLKKIG region, while CAP-18_18_ and CAP-18_14_ had IKEKLKKIGQKIQG structured as a helix, showing folding in the QKIQG region, unlike other variants. The results were further confirmed by the structural alphabet conformations, being predominantly helical across each position of the sequence in PEPFOLD4’s initial template (Appendix B Figure A1).

### 2.6. In Vivo Assays—Toxicity Studies

None of the 11 indicative signs of acute toxicity were observed in the mice groups treated with either CAP-18 or D-CAP-18 peptides. Also, no dermal responses, including erythema/oedema, nor body weight loss were observed during the application at the peptide concentrations used. The mice’s final weight (grams, median ± SD) after 7 days of observation after the treatment were: 18.82 ± 0.25; 19.1 ± 0.55, and 18.9 ± 0.30 for the controls, the CAP-18 or D-CAP-18 peptides, respectively.

### 2.7. Efficacy Studies in Skin Murine Model by P. aeruginosa

The results show that the CAP-18 and D-CAP-18 peptides significantly reduced the bacterial load of *P. aeruginosa* at the site of infection in a mouse skin infection model, compared with the untreated infected control group. Moreover, the efficacies of both peptides at different timepoints increase with a longer duration of therapy.

**Figure 7:**
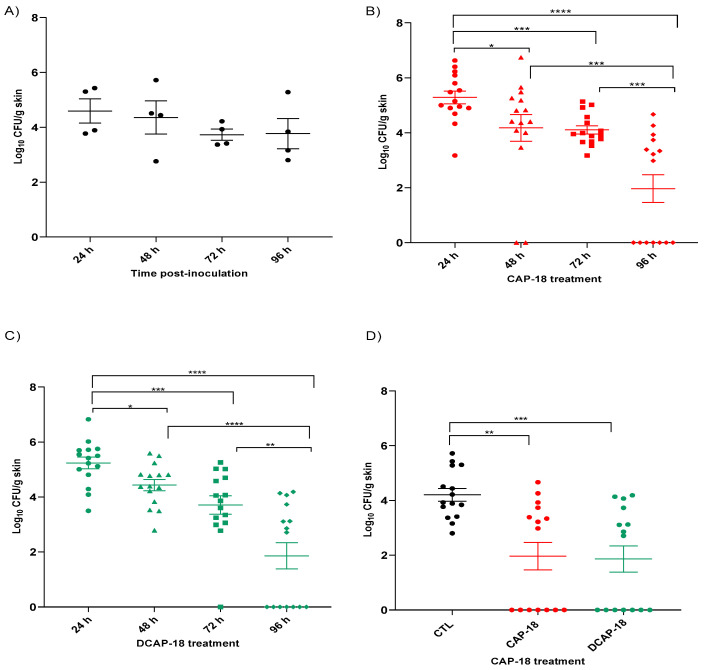
Efficacy of CAP-18 and D-CAP-18 peptides therapy (50 mg/mL/bid/96 h) on skin bacterial concentrations (means ± SEM) of *P. aeruginosa* ATCC 121110. (**A**) Bacterial concentration evolution in the wound area over the course of the 5-day experiment in the untreated infected mice; (**B**) and (**C**) Bacterial concentration evolution in the wound area over the course of 5-day experiment with CAP-18 and D-CAP-18 peptides therapy, respectively, in *P. aeruginosa* ATCC 121110 infected mice; and (**D**) bacterial concentrations in the wound area at the end of experimental therapy with CAP-18 and D-CAP-18 peptides compared with untreated infected mice. Bid: twice daily; CTL: untreated infected mice. * *p* < 0.05; ** *p* < 0.01; *** *p* < 0.001; **** *p* < 0.0001.

The *P. aeruginosa* skin infection was maintained stable throughout the experiment time in the untreated infected control group, with a number of CFU/g dropping of less than 1 log_10_ (Figure 7A). When we compared the bacterial concentration reduction in the CAP-18 treatment (Figure 7B) at different timepoints, we observed that at 48 h, 72 h, and 96 h with this therapy, it significantly reduced the bacterial counts in the skin compared with a 24 h treatment. Similarly, the 96 h CAP-18 treatment significantly reduced the bacterial load compared to 48 h and 72 h of treatment. Moreover, when we evaluated the bacterial skin load at different efficacy timepoints with the D-CAP-18 peptide (Figure 7C), treatment longer than or equal to 48 h significantly reduced the bacterial count in the skin compared to the 24 h treatment. Similarly, the 96 h D-CAP-18 therapy significantly improved the 48 h and 72 h treatment. Finally, regarding the comparison with the control group, both therapies with CAP-18 and D-CAP-18 peptides significantly reduced the bacterial concentration by two orders of magnitude (−2.15 and −2.26 log_10_ CFU/g skin) after 96 h of treatment (Figure 7D).

## 3. Discussion

In this study, we aimed to optimize the antimicrobial activity of the cathelicidin-derived peptide CAP-18 through a combination of in vitro and in vivo approaches. Rational design and activity screening are well established strategies in the development of therapeutic antimicrobial peptides (AMPs) and have previously been applied to shorter fragments of CAP-18 [46,47]. Larrick et al. evaluated CAP-18 derivatives with truncated N- or C-termini for activity against Gram-positive and Gram-negative bacteria, finding that only a 32-amino acid C-terminally truncated peptide retained antimicrobial activity, while other derivatives were inactive [46]. These findings, together with ours, suggest that antimicrobial activity depends not only on amino acid sequence but also on the preservation of secondary structure. The high MIC values of truncated variants discourage their continued development, highlighting the importance of conserved regions, particularly those contributing to structural integrity and positive charge. Consistent with previous reports, our data show that N-terminal truncations of more than five amino acids significantly reduce antimicrobial activity [36,40], as seen in the lower efficacy of CAP-18_23_ compared to CAP-18_31_. Although CAP-18_23_ and CAP-18_21_ exhibited lower activity than other analogs in this study, further optimization through rational design could improve their potency, particularly by enhancing amphipathicity and α-helical content [47]. Further biophysical studies are necessary to obtain a deeper sequence–structure–function relationship and would provide a valuable mechanistic insight beyond our current in silico and microbiological data.

Given that our study focuses on the activity of CAP-18-derived peptides against *P. aeruginosa*, it is pertinent to compare their efficacy to other AMPs reported in the literature. AMPs targeting *P. aeruginosa* are of particular interest due to the pathogen’s clinical relevance and drug resistance. LL-37, a well-known human cathelicidin [48], has shown activity against *P. aeruginosa* [49,50]. A 17-mer LL-37 derivative, RP557, recently demonstrated a MIC of 32 mg/L against *P. aeruginosa* [51], at least 5–6 times higher than the MIC_90_ of all three CAP-18-derived peptides reported in our study. Additionally, chimeric peptidomimetics composed of β-hairpin peptides linked to polymyxin- and colistin-like macrocycles exhibited strong activity against colistin-resistant *P. aeruginosa* strains, with MIC_90_ values between 0.25 and 0.5 mg/L [52]. Two truncated AMPs derived from *Acanthopagrus schlegelii*’s hepcidin, NoAS-hepc3(41–71) and AS-hepc3(48–56), had MICs of 8 µM [53], while rSparanegtin, a recombinant AMP from the mud crab *Scylla paramamosain*, showed MICs ranging from 12 to 24 µM [54]. In comparison, our CAP-18 derivatives displayed MIC_90_ values ranging from 0.45 to 4.3 µM, demonstrating superior potency to many previously reported AMPs.

In addition to antimicrobial efficacy, we evaluated the hemolytic and cytotoxic profiles of our peptides. Compared to OMN6, which showed no hemolytic activity in mouse erythrocytes across a concentration range of 27–868 mg/L (6.25–200 µM) [55], our peptides exhibited higher hemolytic activity. Similarly, AS-hepc3(48–56) preserved 99% erythrocyte integrity after 1 h at 512 µM [53]. However, it is important to note that these studies were conducted on mouse erythrocytes, which differ from human erythrocytes in key membrane characteristics such as sialic acid content, potentially influencing the binding of cationic peptides [56,57]. Moreover, our hemolysis assay involved a longer 4 h incubation period. At 64 mg/L, mastoparan induced 6.6% hemolysis, whereas CAP-18_31_ and D-CAP-18 induced only 4.4% and 3.7%, respectively [58], indicating lower cytotoxic potential.

In terms of cytotoxicity, mastoparan’s IC_50_ against HeLa cells was reported at 32 µM [58], while the guanidylated analog (Gu-INLKALAALAKKIL-NH_2_) had an IC_50_ of 13 µM, comparable to CAP-18_31_. The mastoparan enantiomer (H-inlkalaalakkil-NH_2_) had an IC_50_ of 10 µM, which was less toxic than D-CAP-18 (IC_50_ = 2.11 µM) and D-CAP-18_31_ (IC_50_ = 6.16 µM), though similar to the most toxic mastoparan analog, H-INLKALAALAKKIL-CH_2_CH_2_NH_2_ (IC_50_ = 5 µM). While D-peptides typically exhibit resistance to proteolysis, their enhanced stability does not inherently equate to reduced toxicity.

Time-kill assays further supported the rapid bactericidal activity of our peptides. Although our experiments did not assess earlier timepoints (e.g., <2 h), we hypothesize that a similar fast-acting mechanism exists, particularly at higher concentrations (4× or 8× MIC), as is the case with AS-hepc3(41–71) and AS-hepc3(48–56), which exhibited strong bactericidal effects at 30 and 60 min at 16 µM against *P. aeruginosa* PAO1 [53]. Future membrane permeability studies with finer time resolution (5–10 min intervals up to 1 h) would help clarify the kinetics of action on membrane disruption.

Transmission electron microscopy (TEM) revealed substantial bacterial membrane damage, supporting the hypothesis of membrane-targeting action. Although TEM is a qualitative technique, our observations align with established knowledge of cathelicidin activity [46]. Together, the data suggests that CAP-18 and its derivatives likely interact with lipopolysaccharide (LPS), destabilizing bacterial membranes, a common mechanism among AMPs. This membrane-targeting activity may also be influenced by peptide secondary structure. Previous studies have shown that AMPs adopt stable α-helical conformations upon membrane interaction, contributing to their antimicrobial effect [39]. Our data support this, as CAP-18 and its derivatives exhibit helical structures that likely facilitate their bactericidal activity. However, other structural and physicochemical factors likely influence their selectivity and killing mechanisms.

Finally, we demonstrated the in vivo efficacy of CAP-18-derived peptides using a superficial skin infection mouse model. Most previous studies have utilized burn wound infection models to assess AMP efficacy against *P. aeruginosa*. In contrast, our model better represents non-burn skin infections. A comparable study using Scyreptin1–30, a cationic AMP, reported a significant reduction in bacterial load in a burn wound model [59]. Another study evaluated a modified peptide, PaP1, derived from the lysin PlyPa01, showing effective treatment of burn wounds infected with MDR *P. aeruginosa* both as monotherapy and in combination with gentamicin [60].

CAP-18 and D-CAP-18 peptides show promise as topical antibacterial agents for treating *P. aeruginosa* skin infections. Looking forward, future studies should investigate potential synergistic effects between these peptides and existing antipseudomonal antibiotics in vivo models. Such work will help determine their clinical relevance and therapeutic potential, building on the encouraging results presented here.

## 4. Materials and Methods

### 4.1. Peptides and Peptide Synthesis

The following peptides were purchased as follows: CAP-18, r-CRAMP and Mundticin (InnoPep, San Diego, CA, USA); CRAMP and Dermicidin (Anaspec, Fremont, CA, USA); CRAMP 1-39 and α-defensin 2 (Biosynth, Berkshire, UK); α-defensin 1, α-defensin 3 and α-defensin 6 (PeptaNova GmbH, Sandhausen, Germany); α-defensin 5 (Genaxxon Bioscience GmbH, Ulm, Germany); β-defensin 2 (Hycult Biotech, Madrid, Spain); β-defensin 3 and β-defensin 4 (Abbexa, Cambridge, UK); Hepcidin (SB-PEPTIDE, Saint Egrève, France); HP 2-20 (Innovagen, Lund, Sweden); PR39 (Enzo Life Sciences, Farmingdale, NY, USA). After selecting CAP-18 as a peptide to work with, the first CAP-18 peptide was synthesized at Institut de Recerca Biomèdica (IRB), Barcelona, Spain. Peptides CAP-18_31_, CAP-18_21_, CAP-18_23_, CAP-18_19_, CAP-18_18,_ and CAP-18_14_ were synthesized by Iproteos, Barcelona, Spain. Peptides D-CAP-18, D-CAP-18_31_, R-CAP-18, and R-CAP-18_31_ were synthesized by GenicBio (Shanghai, China).

CAP-18 (MW = 4433.48) was synthesized through solid phase synthesis. CAP-18 was synthesized on the CEM Liberty Blue™ (Matthews, NC, USA) Automated Microwave-assisted Peptide Synthesizer at a 0.10 mmol scale using Wang resin preloaded with Tyr (1.03 mmol/g substitution). The resin pre-loading was performed manually on the Wang resin through the ester bond formation. 0.516 mmol of resin (1.03 mmol/g substitution) was swelled in DCM (45 min). The coupling was performed in DCM using 4 eq Fmoc–Tyr(tBu)–OH, 4 eq DIC, and 0.4 eq DMAP for 30 min and repeated three times. The new loading was determined (1.03 mmol/g).

To allow the chain growth, a series of deprotection, coupling, and washing steps was performed. 20% *w*/*v* piperidine in DMF with 0.1 M Oxyma was used for Fmoc deprotection. Post-deprotection washing with DMF (3 × 2 mL) was followed by coupling using a 5-fold excess of reagents: Fmoc–AA–OH (0.2 M in DMF, 2.0 mL), DIC (0.5 M in DMF, 1.0 mL), and Oxyma (1.0 M in DMF with 0.1 M DIPEA, 0.5 mL). After each coupling, the resin was washed with DMF (5 × 3 mL). Several amino acids were double coupled (in bold): **G**-**L**-**R**-K-**R**-**L**-**R**-**K**-**F**-**R**-N-**K**-**I**-**K**-E-**K**-**L**-K-**K**-**I**-G-Q-**K**-**I**-Q-**G**-**L**-**L**-P-**K**-**L**-A-**P**-**R**-T-D-Y. After synthesis, the resin was rinsed with DMF (3 × 5 mL), DCM (3 × 5 mL), and diethyl ether (5 × 5 mL). The peptide was cleaved from the resin (4 h) with TFA/TIS/H2O (95/2.5/2.5) and precipitated in cold ether. The obtained crude material was analyzed without any purification (92% pure). Next, the peptide was purified using preparative HPLC. Crude peptides were analyzed on a Waters UPLC H-class (Milford, MA, USA) coupled to an electrospray ion source ESI-MS Micro mass ZQ, using acetonitrile/water with 0.1 % TFA (gradient: 0–100% acetonitrile) as the solvent system on a BEH C18-column (internal diameter, 2.1 mm; length, 50 mm; particle size, 1.7 μm). Detection was at 214 nm.

Peptides were purified by semi-preparative HPLC on a Waters 2700 sample manager equipped with a Waters 2487 dual-wavelength absorbance detector, a Waters 600 controller, a Waters fraction collector and Masslynx software v4.1 by using a Sunfire C18 column (150 × 10 mm × 3.5 μm, 100 Å, Waters), flow rate 6.6 mL/min, solvent A = 0.1% trifluoroacetic acid in water; solvent B = 0.1% trifluoroacetic acid in acetonitrile (gradient: 10–45% B).

**Figure A2:**
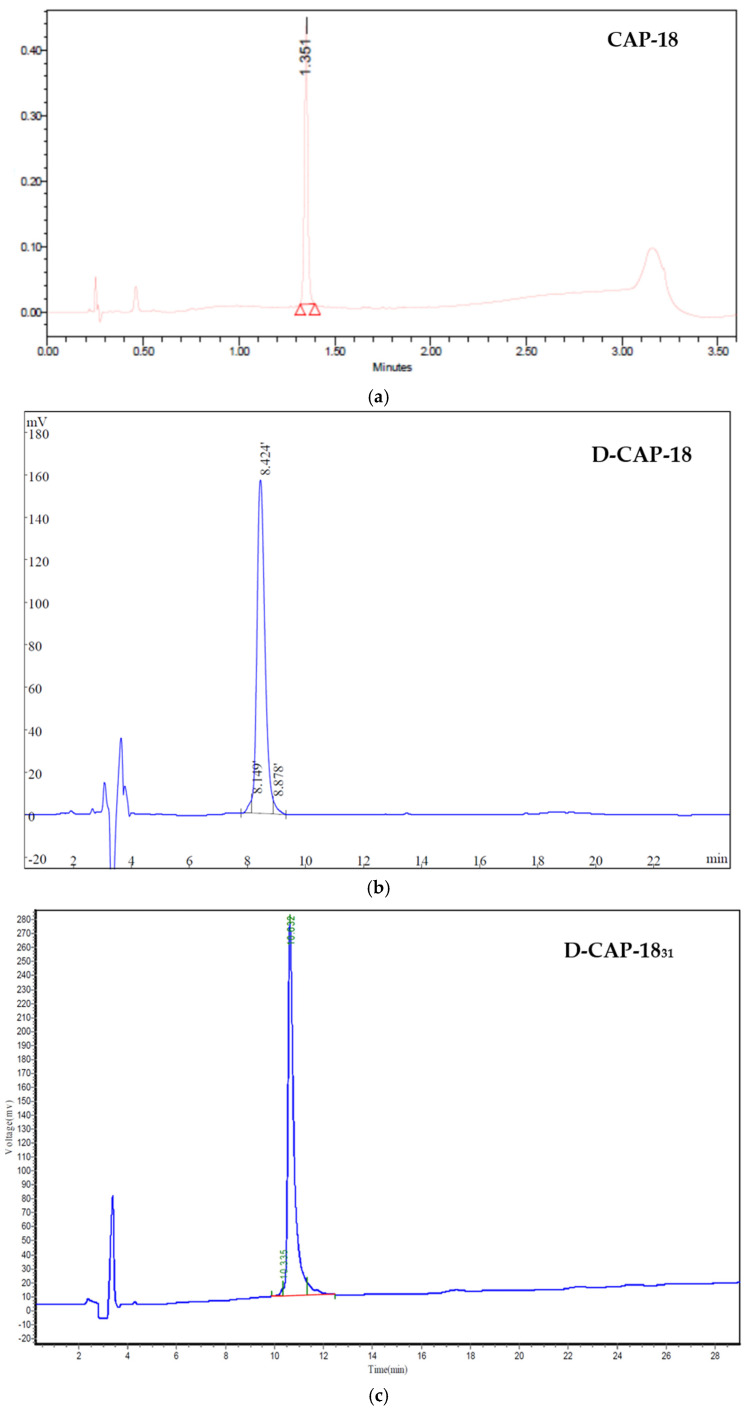

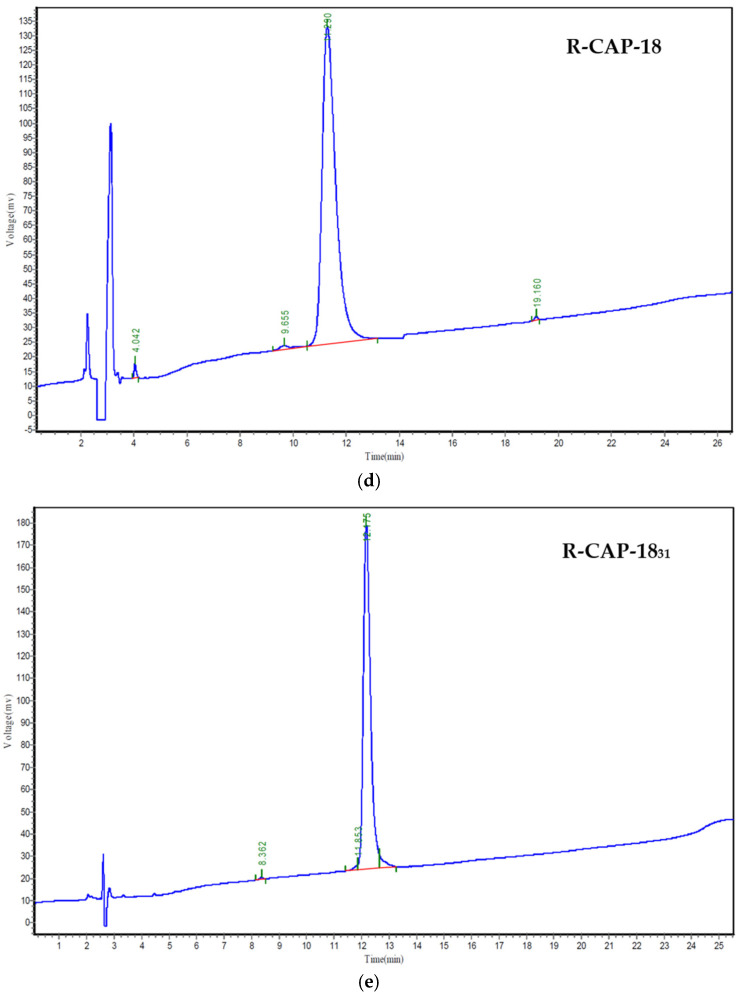
UPLC chromatograms of peptides (**a**) CAP-18 and HPLC chromatograms of peptides, (**b**) D-CAP-18, (**c**) D-CAP-18_31_, (**d**) R-CAP-18; and (**e**) R-CAP-18_31_ after purification.

Peptides were characterized by either HPLC or UPLC. Peptide CAP-18 (Figure A2a) was analyzed on a UPLC Agilent system (Santa Clara, CA, USA, equipped with an Acquity BEH C18, 2.1 × 50 mm column), using a 20–80% B gradient (solvents A = 0.045% TFA in water, and B = 0.036% TFA in acetonitrile). Peptides D-CAP-18, D-CAP-18_31_, R-CAP-18, and R-CAP-18_31_ were analyzed by HPLC in a Shimadzu instrument (Kyoto, Japan) equipped with a Kromasil C18 4.6 × 250 mm column, using a 30–55% B gradient (solvents A = 0.1% TFA in water, and B = 0.1% TFA) over 25 min.

**Figure A3:**
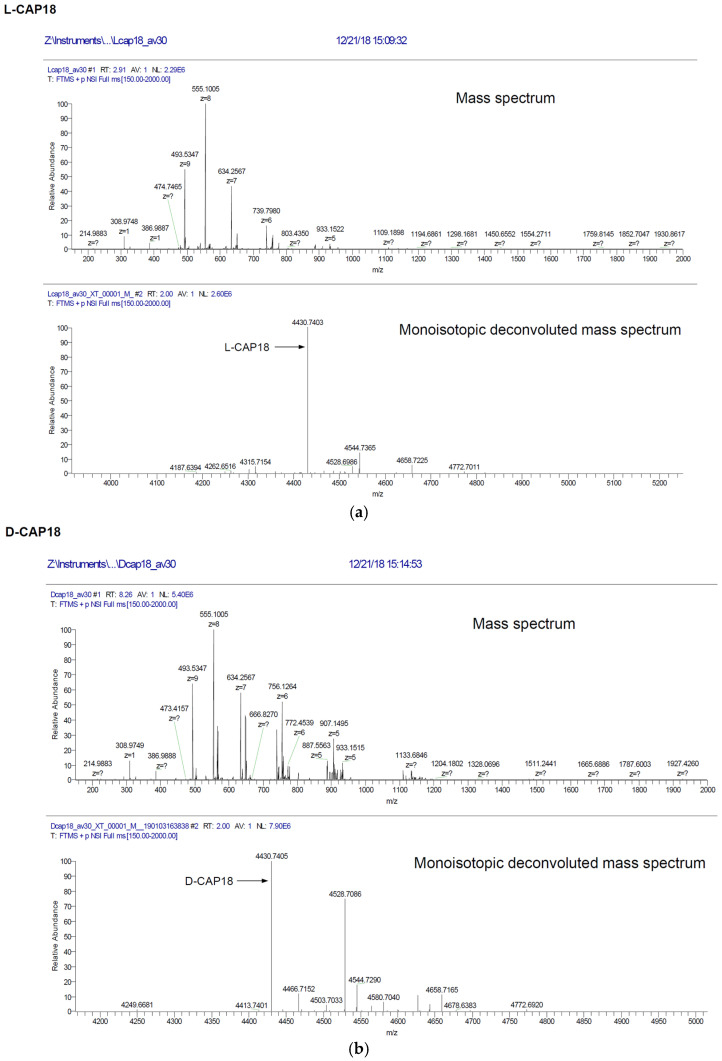

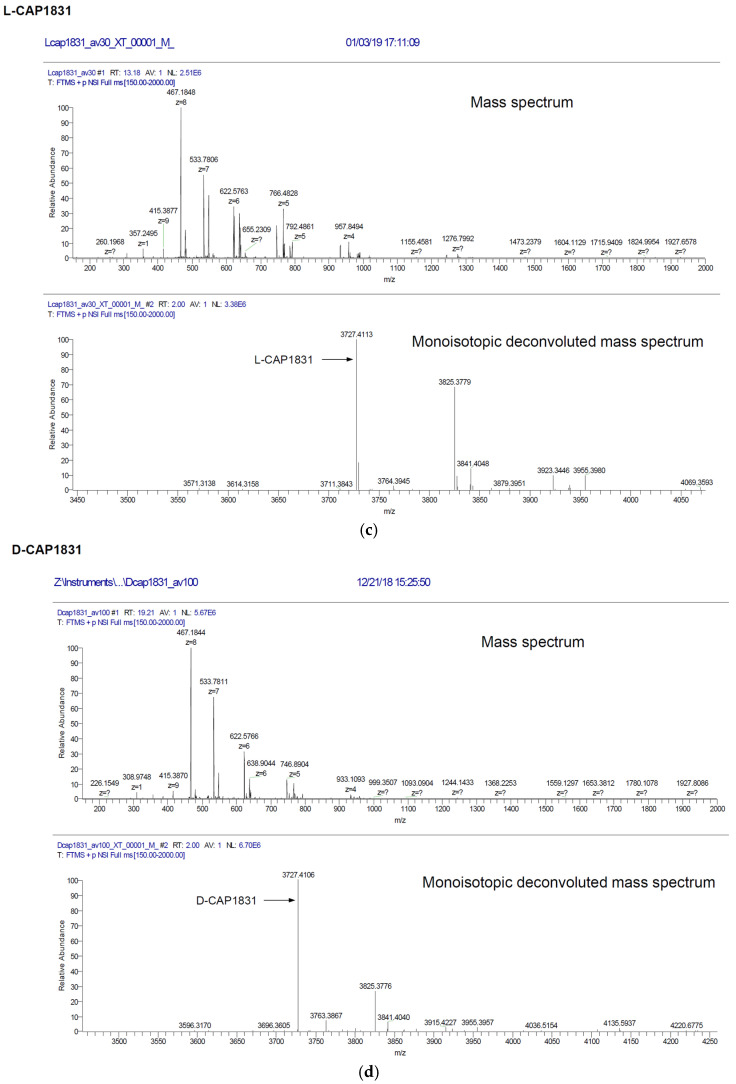
High-resolution mass spectrometry characterization data for (**a**) CAP-18 (L-CAP-18), (**b**) D-CAP-18, (**c**) CAP-18_31_ (L-CAP-18_31_); and (**d**) D-CAP-18_31_. (z = charge number of ions).

High-resolution mass spectrometry characterization data for CAP-18, CAP-18_31_, D-CAP-18, and D-CAP-18_31_ is provided in the Appendix C (Figure A2 and Figure A3).

### 4.2. Bacterial Strains

The antimicrobial activity of the peptides was tested against a collection of *P. aeruginosa*, selected by their resistance or susceptibility to colistin and/or classified as MDR strains. The origin of the strains was either clinical, relevant to the current clinical situation, or commercial strains belonging to the American Type Culture Collection (ATCC, Manassas, VA, USA), which complemented the collection for comparison and control. For the initial activity screening of the 17 peptides, we tested them against *P. aeruginosa* ATCC strains 1211007 and 1211101. Both strains are multidrug-resistant; however, the key difference is that strain 1211007 is susceptible to colistin, whereas strain 1211101 is resistant to colistin.

### 4.3. Antimicrobial Susceptibility Testing

Minimum Inhibitory Concentration (MIC) of the antimicrobial agents against bacterial strains was obtained through broth microdilution assays following a protocol based on the Clinical & Laboratory Standards Institute’s guidelines [61]. Modifications regarding microplate material and broth were included when testing the peptides’ activity and are detailed below. Colistin MICs were obtained following recommendations of the joint CLSI-EUCAST Polymyxin Breakpoints Working Group using Cation Adjusted Mueller-Hinton Broth (90922, 500 g Sigma-Aldrich^®^, St. Louis, MO, USA), and strains *Escherichia coli* 13846 (*mcr-1* positive) and *P. aeruginosa* ATCC 27853 were used as quality controls in all assays. Bacteria were cultured in Columbia Agar plates (Columbia Agar with 5% Sheep Blood 90 mm Stacker plates, Becton Dickinson, Franklin Lakes, NJ, USA) and incubated overnight at 37 °C. Polypropylene round-bottomed 96-well plates (-96-well Polypropylene Storage Microplates, 267334, Thermo Fischer Scientific, Waltham, MA, USA) were used to prevent peptide binding to plate walls. AST broth (BD Phoenix™ AST broth, 8 mL, 246003, Becton Dickinson, Franklin Lakes, NJ, USA) was used when testing the peptides’ activity. MIC values were determined after 18 to 22 h incubation at 37 °C. Three biological and technical replicates were performed per strain.

### 4.4. Hemolysis Assays

Commercially available human blood (IWB1K2E10ML, Innovative Research Inc., Novi, MI, USA) was used to produce a 50% haematocrit solution by mixing at 1:1 with PBS 1× (P5493, 1 L, Sigma, St. Louis, MO, USA). Three consecutive PBS 1× washes plus centrifugation at 2600 rpm for 10 min steps were performed. Finally, a 2% erythrocyte solution was prepared in PBS 1× and chilled on ice until used. Polypropylene plates were filled with 50 µL PBS 1× per well for the samples and 50 µL 2% Triton X-100 (TX-100, 9002-93-1, 100 mL, Sigma, St. Louis, MO, USA) for the hemolysis controls. A solution at 4× final concentration of the assay of each peptide was prepared in PBS and 50 µL were added to the first column of each plate, followed by serial dilutions in the microplate. Then, 50 µL of the 2% erythrocyte solution was added to each plate. Positive controls consisted in a 1:1 mix of PBS 1× and erythrocytes at 2%. Plates were incubated at 37 °C for 4 h and then centrifuged at 1500 rpm for 5 min. Eighty µL were extracted from the supernatant of each well carefully and transferred to a polystyrene flat bottom plate for reading and read at 450 nm in an Epoch microplate spectrophotometer (Epoch™, BioTek, Winooski, VT, USA).

The therapeutic index is a widely accepted parameter to represent the specificity of antimicrobial peptides for prokaryotic versus eukaryotic cells. It is calculated by the ratio of HC50 (hemolytic activity) and MIC (antimicrobial activity); thus, larger values of therapeutic index indicate greater specificity for prokaryotic cells.

### 4.5. Cytotoxicity Assays

Cell viability assays were performed against HeLa and A549 cells. Cells were seeded in Corning 96-well microplates at 2 × 10^3^ cells/well and 5 × 10^3^ cells/well and incubated for 24 h at 37 °C. Then, cells were treated with peptides dissolved in Dulbecco’s Modified Eagle’s Medium for 24 h; peptide concentration range 0.1–100 µM. The peptide solution was then removed, and fresh medium was added to the cells. Finally, 50 µL of activated-XTT solution was added and incubated for 4 h at 37 °C. A PowerWave X reader (BioTek, Winooski, VT, USA) was used to measure absorbance at 450 nm. Cell viability was calculated through the ratio of the absorbance of peptide-treated cells over the absorbance of untreated cells. Triplicates were run for each experiment.

### 4.6. Time-Kill Kinetic Assays

Time-kill curves were designed to test MIC, 2× MIC, 4× MIC, aWinooskind 8× MIC based on MIC data from peptides CAP-18, CAP-18_31_, D-CAP-18, and D-CAP-18_31_ against the strains *P. aeruginosa* R2 and 121110. Timepoints to withdraw aliquots of liquid cultures for each peptide, concentration, and strain were established at the following: 0, 2, 4, 8, and 24 h after inoculation. McFarland 0.5 solutions (~1.5 × 10^8^ CFU/mL) were prepared from single-picked colonies grown O/N at 37 °C in Columbia Agar plates. Thirteen mL Falcon tubes were used to prepare solutions containing 5 mL of total volume by adding the following: 1 mL of peptide solution at 5 times chosen assay concentration to test, 3.75 mL AST, and 0.25 mL of a 1:10 McFarland dilution in AST. Positive controls contained 4.75 mL AST and 0.25 mL of strain inoculum diluted in AST; negative controls only contained 5 mL of AST. After adding the inoculum into each tube and vortexing, a 150 µL aliquot was taken from each tube at timepoint 0 h. All tubes were then placed in a 37 °C shaker-incubator at 180 rpm. Likewise, for timepoints 2, 4, 8, and 24 h, a 150 µL aliquot was withdrawn at each time for each tube. Each of these aliquots was serially diluted in 1:10 ratio in PBS 1× and 20 µL of the serial dilutions were spread on LB agar plates and incubated O/N at 37 °C. O/N. C colonies grown in LB agar plates were counted. Bacterial concentration (CFU/mL) for each strain, timepoint, and peptide concentration tested was calculated. Bactericidal effect was considered when there was a decrease of at least 3 in log_10_ (CFU/mL) for each peptide concentration at a given timepoint against each bacterial strain studied. A detection limit of 50 CFU/mL was established, corresponding to 1.70 log_10_ (CFU/mL), as 20 µL of each direct aliquot sample was spread in LB agar plates. Three replicates were run per peptide and strain.

### 4.7. Transmission Electron Microscopy Sample Visualization

Bacterial strains *P. aeruginosa* R2 and 121110 under treatment with CAP-18, CAP-18_31_, D-CAP-18, and D-CAP-18_31_ were chosen for TEM visualization. A total of 5 mL AST broth cultures were prepared in 50 mL Falcon tubes and inoculated with single colonies grown in Columbia Agar incubated at 37 °C. Liquid cultures were then diluted 1:100 in fresh AST broth and incubated at 37 °C until OD 0.6 was reached. Cultures were then centrifuged 10 min at 3000× *g* at 4 °C, and supernatant was discarded and replaced by fresh AST. Fifty mL Falcon tubes were filled up to 5 mL with 4 mL of 0.6 OD inoculum and 1 mL of peptide treatment at 4 or 8× MIC and incubated at 37 °C for either 2 or 4 h. Since no differences were observed in the TEM analysis, the condition of 4× MIC for 4 h was selected and is presented in the Section 2. Results from TKC assays were used as a guidance to choose peptide concentrations and incubation times for TEM sample visualization. After incubation, tubes were centrifuged thrice for 15′ at 5000× *g* and 4 °C, substituting supernatant after each centrifugation by adding 10 mL PBS 1× to the pellet and resuspending. Supernatant was discarded after the final centrifugation step. Pellet was fixed by adding 1 mL of 2.5% glutaraldehyde + 2% paraformaldehyde in PBS 0.1 M at pH 7.4, and centrifuged 10′ at 5000× *g* and 4 °C. Supernatant was discarded 30 min after centrifugation and pellet was washed twice with 1 mL of fresh fixing solution. Samples were stored 4 °C O/N and processed by the Electronic Microscopy Unit, Medicine Faculty, University of Barcelona. University of Barcelona Microscopy Facilities.

### 4.8. Secondary Structure Prediction

The secondary structure of peptides in physiological conditions is difficult to simulate due to current molecular dynamics packages not being sufficiently optimized for such a task and for the computational cost they require. For the estimation of the secondary structure of peptides, the open-source PEPFOLD4 was used [45,62,63,64]. Peptide sequences written in the FASTA format were used as input and inserted into PEPFOLD4 (available at: https://mobyle2.rpbs.univ-paris-diderot.fr/cgi-bin/portal.py#forms::PEP-FOLD4, accessed on 30 July 2024). The structural alphabet profile sampler was set to taboo-sampling [63]. The number of Monte-Carlo steps was set at 30,000 with a temperature of 370 K. To have reproducible results, a pseudo-random seed was set to one instead of a random seed. The Debye–Hückel protocol was used to predict the structures at pH 7.5 and a NaCl concentration of 150 mM with the N- and C-terminus remaining charged. These conditions were chosen to be the closest to physiological conditions. The structural alphabet mentioned here is a library of 27 protein backbone conformations for each combination of four residue length fragments [65].

To analyze the structures, the five best-scoring PDB files were downloaded for each sequence and analyzed in Visual Molecular Dynamics (VMD) software (v1.9.3), where the label option and ‘NewCartoon’ preset showed where the helix started and ended.

### 4.9. In Vivo Assays

Immunocompetent female BALB/C mice weighing 20 g (7–9 weeks old) were used (Charles River laboratories, St-Germain-Nuelles, France). Animals were murine pathogen-free and were assessed for genetic authenticity. The study followed the Guide for the Care and Use of Laboratory Animals [66]. The experiments followed the 2010/63/EU directive on the protection of animals used for scientific research. Experiments were approved by the Committee on the Ethics of Animal Experiments of the Virgen del Rocío University Hospital and the Andalusian Ministry of Agricultura, Pesca, Agua y Desarrollo Rural, Junta de Andalucía, Sevilla, Spain (11-09-15-322).

### 4.10. Toxicity Studies

CAP-18 and D-CAP-18 peptides were evaluated in five healthy female BALB/C mice at the dosage and regimen schedule to be used in the efficacy studies. The following indicative signs of acute toxicity were assessed during seven days: reduced water (dehydration)/food intake, isolation, self-mutilation, tremors/spasms, dyspnea, physical activity (increased/reduced), chromo-dacryorrhea, dermal responses, including erythema/oedema/redness/discoloration/necrosis), muscle stiffness, piloerection, teeth grinding, and weight loss.

### 4.11. Efficacy Studies in Skin Murine Infection Model with P. aeruginosa

A previously characterized skin model infection was used [67]. Briefly, groups of sixty mice were randomly included in the following topically therapeutic groups: (i) controls (infected, untreated); (ii) CAP-18 (50 mg/mL/bid/96 h); and (iii) D-CAP-18 (50 mg/mL/bid/96 h). Animals were intraperitoneally (ip) anesthetized (ketamine/diazepam). Then, an approximately 2 cm^2^ area was shaved, the exposed skin was disinfected (80% ethanol), and a superficial wound was gently made with a sterile metal scalpel. Next, this wounded area was inoculated with 20 µL of 8.76 log_10_ CFU/mL *P. aeruginosa* ATCC 121110. Once the inoculated wound was dried, it was covered with a sterile gauze. Treatment was initiated 24 h post-inoculation and lasted 96 h. Fifteen mice per group were sacrificed after 24, 48, 72, and 96 h post-infection. The wounded area was aseptically removed and processed for quantitative cultures (log_10_ CFU/g). The groups of animals were completed in different weeks until the sample size per timepoints and treatment groups was reached.

### 4.12. Statistical Analysis

Bacterial concentrations are expressed as means ± SEM. Differences in bacterial concentrations between groups were compared by the Mann–Whitney test. A *p* value < 0.05 was considered significant. The SPSS v22.0 software was used (SPSS Inc., Chicago, IL, USA).

## 5. Conclusions

We are already in urgent need of new antibiotics, particularly those effective against multidrug-resistant (MDR) bacteria such as *Pseudomonas aeruginosa*. Antimicrobial peptides (AMPs) have emerged as a promising alternative strategy for treating infections caused by resistant pathogens. In this study, derivatives of CAP-18 were synthesized and screened for antimicrobial activity against MDR *P. aeruginosa* strains.

Among the shorter CAP-18 derivatives, only CAP-18_31_ retained activity against *P. aeruginosa*. Additionally, the retroenantio version of CAP-18 exhibited reduced antimicrobial activity. Based on these findings, we selected CAP-18, CAP-18_31_, D-CAP-18, and D-CAP-18_31_, the most active compounds, for further evaluation.

All four peptides demonstrated bactericidal activity within short incubation times (2 to 8 h) against *P. aeruginosa* strains R2 and 121110. D-CAP-18_31_ showed the lowest toxicity toward human erythrocytes, while D-CAP-18 displayed the highest therapeutic index. The mechanism of action appears to involve membrane permeabilization. Furthermore, both D-CAP-18 and D-CAP-18_31_ exhibited strong in vivo antimicrobial activity.

Based on our screening and results, we consider CAP-18, D-CAP-18, CAP-18_31_, and D-CAP-18_31_ to be promising candidates for the topical treatment of skin infections caused by MDR *P. aeruginosa*, particularly colistin-resistant strains. Among these, D-CAP-18 stands out as the most effective peptide, with superior in vitro activity, a higher therapeutic index, and favorable in vivo efficacy.

## 6. Patents

The results arising from this project are pending patentability analysis by patent attorneys.

## Figures and Tables

**Table 1:** Activity of different peptides against colistin-susceptible and colistin-resistant strains of *P. aeruginosa*.

	MIC (mg/L) [μM]	
Peptide	Colistin Susceptible *P. aeruginosa*	Colistin-Resistant *P. aeruginosa*
r-CRAMP	>256 [>65]	64 [16]
α-defensin 3	>50 [>14]	>50 [>14]
α-defensin 1	>50 [>15]	>50 [>15]
PR39	64 [14]	32 [7]
β-defensin 4	>50 [>11]	>50 [>11]
CRAMP	256 [>66]	32 [8]
β-defensin 2	>50 [>12]	>50 [>12]
α-defensin 6	>50 [>13]	>50 [>13]
β-defensin 3	>50 [>10]	>50 [>10]
Dermicidin	>256 [>53]	>256 [>53]
Mundticin	>256 [>60]	>256 [>60]
CRAMP 1-39	64 [>14]	32 [7]
α-defensin 2	>50 [>15]	>50 [>15]
**CAP-18**	**8 [1.8]**	**4 [0.9]**
HP 2-20	>256 [>110]	>256 [>110]
Hepcidin	>256 [>92]	>256 [>92]
α-defensin 5	50 [14]	>50 [14]
Colistin	0.75 [0.7]	32 [28]

CAP-18 and the numbers in bold refer to the peptide that was selected for further studies from the screened peptides since it was the one showing better activity against *P. aeruginosa*.

**Table 2:** Definition, sequences, and MIC of CAP-18 and derived peptides used in the study.

		MIC (mg/L) [μM]	
Peptide	Sequence	Strain 121007	Strain ATCC 121110
CAP-18	GLRKRLRKFRNKIKEKLKKIGQKIQGLLPKLAPRTDY	1 [0.2]	<0.125 [<0.03]
CAP-18_31_ *	GLRKRLRKFRNKIKEKLKKIGQKIQGLLPKL	8 [2.1]	0.5 [0.1]
CAP-18_23_ *	FRNKIKEKLKKIGQKIQGLLPKL	>64 [>24]	>32 [>12]
CAP-18_21_ *	GLRKRLRKFRNKIKEKLKKIG	32 [12]	32 [12]
CAP-18_19_ *	IKEKLKKIGQKIQGLLPKL	>64 [>29]	>64 [>29]
CAP-18_18_ *	FRNKIKEKLKKIGQKIQG	>64 [>30]	>64 [>30]
CAP-18_14_ *	IKEKLKKIGQKIQG	>64 [40]	>64 [>40]
D-CAP-18	Glrkrlrkfrnkikeklkkigqkiqgllpklaprtdy	2 [0.4]	0.25 [0.06]
D-CAP-18_31_	Glrkrlrkfrnkikeklkkigqkiqgllpkl	2 [0.5]	0.25 [0.07]
R-CAP-18	Ydtrpalkpllgqikqgikklkekiknrfkrlrkrlg	2 [0.4]	32 [7.2]
R-CAP-18_31_	Lkpllgqikqgikklkekiknrfkrlrkrlg	0.5 [0.1]	4 [1.1]

Minor cases show D-amino acids. Peptides marked with (*) were used as crudes.

**Table 3:** MIC_50_ and MIC_90_ values of *P. aeruginosa* strain panel against CAP-18, CAP-18_31_, D-CAP-18, D-CAP-18_31_, and colistin.

	*P. aeruginosa* Strain Panel	
Peptide	MIC_50_ (mg/L) [μM]	MIC_90_ (mg/L) [μM]
CAP-18	0.5 [0.1]	4 [0.9]
CAP-18_31_	2 [0.5]	16 [4.3]
D-CAP-18	1 [0.2]	2 [0.45]
D-CAP-18_31_	4 [1.1]	16 [4.3]
Colistin	≤0.125 [≤0.11]	64 [55]

**Table 4:** Peptide hemolysis, cell viability IC_50_ values, and ratio between hemolysis IC_50_ and MIC_90_ of each peptide against the pathogen species tested.

Peptide	Hemolysis IC_50_ (μM) [mg/L]	A549 IC_50_ (µM) [mg/L]	Therapeutic Index (IC_50_/MIC_90_) *P. aeruginosa*
CAP-18	35.73 [158.41]	0.87 [3.86]	39.6
CAP-18_31_	185.26 [690.97]	1.31 [4.89]	43.2
D-CAP-18	61.97 [274.76]	2.09 [9.26]	137.4
D-CAP-18_31_	108.44 [404.48]	12.32 [45.94]	25.3

## Data Availability

All data generated or analyzed during this study are included in this article. Further inquiries can be directed at the corresponding authors.

## Abbreviations

The following abbreviations are used in this manuscript:

AMR	Antimicrobial Resistance
WHO	World Health Organization
MDR	Multidrug resistant
AMP	Antimicrobial peptide
MIC	Minimum Inhibitory Concentration
TKC	Time-kill curve
ATCC	American Type Culture Collection
TEM	Transmission Electron Microscopy
CDR	Carboxyl-terminal disordered region
VMD	Visual Molecular Dynamics

## Appendix A

**Table A1:** MIC results for *P. aeruginosa* strains.

	MIC (mg/L)				
*P. aeruginosa* Strains	Colistin	CAP-18	CAP-18_31_	D-CAP-18	D-CAP-18_31_
**12-1110 S1**	≤0.125	≤0.125	0.5	0.25	0.25
**21-0505 S2**	≤0.125	≤0.125	0.5	0.25	0.5
**21-0308 R2**	64	2	8	2	4
**15-0803 S3**	≤0.125	0.5	2	0.25	4
**15-0307 S4**	≤0.125	≤0.125	0.5	≤0.125	0.5
**21-0410 S5**	≤0.125	0.25	1	0.5	0.5
**16359347-2 R3**	64	1	8	2	4
**36a**	0.25	0.5	1	1	2
**38a**	≤0.125	1	4	1	4
**27853**	≤0.125	1	4	2	4
**C1**	0.25	4	16	2	16
**C2**	0.5	0.25	16	1	16
**C3**	≤0.125	0.25	2	2	8
**C4**	0.25	4	4	1	8
**C5**	≤0.125	1	2	1	4

## Appendix C

**Table A2:** Peptide sequences, molecular weight, and molecular formula of the synthesized peptides in this study.

Peptide	Sequence	Formula	MW (Da)
CAP-18	GLRKRLRKFRNKIKEKLKKIGQKIQGLLPKLAPRTDY	C_202_H_356_N_64_O_47_	4433.45
CAP-18_31_	GLRKRLRKFRNKIKEKLKKIGQKIQGLLPKL	C_171_H_311_N_55_O_37_	3729.70
CAP-18_23_	FRNKIKEKLKKIGQKIQGLLPKL	C_127_H_226_N_36_O_29_	2721.42
CAP-18_21_	GLRKRLRKFRNKIKEKLKKIG	C_118_H_217_N_41_O_25_	2610.28
CAP-18_19_	IKEKLKKIGQKIQGLLPKL	C_102_H_187_N_27_O_24_	2175.77
CAP-18_18_	FRNKIKEKLKKIGQKIQG	C_98_H_174_N_30_O_24_	2156.65
CAP-18_14_	IKEKLKKIGQKIQG	C_73_H_135_N_21_O_19_	1611.00
D-CAP-18	Glrkrlrkfrnkikeklkkigqkiqgllpklaprtdy	C_202_H_356_N_64_O_47_	4433.45
D-CAP-18_31_	Glrkrlrkfrnkikeklkkigqkiqgllpkl	C_171_H_311_N_55_O_37_	3729.70
R-CAP-18	Ydtrpalkpllgqikqgikklkekiknrfkrlrkrlg	C_202_H_356_N_64_O_47_	4433.45
R-CAP-18_31_	Lkpllgqikqgikklkekiknrfkrlrkrlg	C_171_H_311_N_55_O_37_	3729.70
